# Comparison of Psychometric Performance of Faculty and Student Authored Anatomy Exam Items in Medical Education

**DOI:** 10.1007/s40670-026-02694-8

**Published:** 2026-03-07

**Authors:** Shanna Williams, Helen Kaiser, Sydny Long

**Affiliations:** Biomedical Sciences Dept, University of South Carolina School of Medicine Greenville, 607 Grove Rd. , SC 29605 Greenville, USA

## Abstract

Near-peer teaching is an effective method for improving student retention and comprehension in undergraduate medical education. This approach involves students providing content instruction to peers who are one or more years junior. Both the student-teacher and the recipient have been found to benefit from increased self-confidence and understanding of the material in pre-clinical and clinical environments. At the University of South Carolina School of Medicine Greenville (USCSOMG), we implemented an elective rotation for fourth-year medical students (M4s) that included teaching anatomy to first-year students in the dissection lab and writing summative exam questions with faculty review similar to the process faculty undergo. M4s contributed to organ system-centric exams during the 2021–2022 and 2022–2023 academic years. Test items were analyzed to assess their difficulty, discrimination index, and point biserial, comparing questions written by faculty and M4s. A significant difference was found in point biserials of exams 2 C and 2D, (Exam 2 C: Faculty 0.1467, M4 0.2342, *P* < 0.001; Exam 2D: Faculty 0.33, M4 0.1829, *P* = 0.007) and discrimination index of exam 2 C (Exam 2 C: Faculty 0.8525, M4 0.8850, *P* = 0.031). These results highlight the unique strengths both groups bring to the question-writing process: faculty’s advanced content knowledge and experience, and M4s’ recent familiarity with the material. Based on these findings, we propose leveraging upper-class medical students as valuable resources in generating assessment materials. This practice can contribute to a more clinically-relevant curriculum and enhance the educational skills of early-career doctors.

## Introduction

The practice of placing the student in the instructor role (also known as peer-teaching) has been shown to enhance comprehension and knowledge retention in student learners [1–3]. Peer-teaching refers to instruction delivered by students of a similar training level, whereas near-peer teaching involved instruction provided by students who are one of more years ahead in training. Peer-teaching has been shown to be an effective teaching approach in the sciences, where accessible communication of complex and theoretical concepts is crucial to student success [4, 5].

Undergraduate medical education presents difficult concepts to a diverse group of students within a relatively brief time frame compared to undergraduate education [6]. The literature indicates peer-instruction improves student performance both in the biomedical science and clinical components of the curriculum [4, 5]. Two studies examined peer teaching within these two contexts by comparing first-year student performance on a physical exam and a biomedical science formative test [7, 8]. Students were either taught by a faculty member or a fourth-year medical student (colloquially known as an M4) from the same institution, and the studies arrived at similar conclusions: first-year students received near identical scores on the physical patient exam and the traditional assessment regardless of their instructor’s position [7, 8]. Instruction from peers or near-peers not only produces comparable results to those obtained from traditional faculty-led instruction, it also is rated more favorably by participating students on measures of helpfulness and usefulness and shows reductions in feelings of performance anxiety [9, 10]. In addition to these benefits, fourth-year medical students’ comprehension and performance was enhanced through the peer teaching process [7, 8].

While the relationship between student and student-teacher benefits the student, it can also benefit the student-teacher, particularly in the context of medical education in which foundational knowledge must be constantly revisited [6]. Medical students in the clinical phase of their training are preparing to enter residency, a transition that introduces more consistent expectations for teaching others [11]. To support this transition, many medical universities incorporate targeted education workshops and peer-instruction [11]. Integration of teaching opportunities in the clinical curriculum has demonstrated a positive impact on students and early-career physicians by improving performance on Objective Structured Teaching Exercise (OSTE) stations that test clinical skills [12, 13]. A four-hour “teaching to teach” course for graduating fourth-year medical students increased confidence, with 84% of students choosing “agree” or “strongly agree” with the statement: “The teaching to teach course helped prepare me for my role as a teacher during internship” [14].

Peer-teaching as a means of improving teaching skills in fourth-year students has been received favorably by students and improves fourth-year students performance [7, 9, 15]. However, the role of student-teachers in developing summative assessments remains under-examined in the literature [7, 9, 15]. Limited research suggests that drafting multiple-choice exam items and revising items with faculty support increases students’ perceived understanding and application of the material [16, 17]. Teaching relevant content and assisting preclinical students in learning physical procedures and skills also reinforces fourth-year students’ own performance [8]. Despite these findings, little is known about how near-peer constructed items perform on pre-clerkship summative examinations. This study aims to evaluate the efficacy of M4-authored, faculty reviewed anatomy exam questions, compared to faculty- authored items that underwent standard faculty peer review across summative exams in two cohorts of pre-clerkship students. Based on the previous studies showing positive perceptions of near-peer teaching and positive performance on near-peer anatomy practical formatives, we hypothesized that faculty and M4 authored anatomy questions would not differ significantly in their psychometric performance [7, 8].

## Materials and Methods

### Student Population

The study included 208 first-year medical students (M1s) enrolled in gross anatomical sciences courses at the University of South Carolina School of Medicine Greenville (USCSOMG). This comprised the Class of 2025 (63% female, 37% male; ages 23–32, average age 25.6) and the Class of 2026 (56% female, 44% male; ages 22–43, average age 25.1). These cohorts completed the Structure and Function I and II modules during the 2021–2022 and 2022–2023 academic years, respectively. This study was determined “exempt” by the University of South Carolina IRB Committee (Reference #Pro00129373).

### Educational Context

Gross anatomical sciences content was delivered through the Structure and Function (SF) I and II modules, which together constitute over 50% of the first-year medical curriculum. SF I and SF II were offered in consecutive semesters during each academic year. Exam data were drawn from eight summative assessments administered across these modules: four exams in 2021–2022 (Exams 1 A–1D, Class of 2025) and four exams in 2022–2023 (Exams 2 A–2D, Class of 2026). Each exam focused on a specific organ system (A: upper limb musculoskeletal; B: lower limb musculoskeletal and cardiopulmonary; C: abdominal and reproductive; D: head and neck). Exams were delivered via ExamSoft on school-issued iPads, with 84–144 questions per exam and a time limit of 105 s per question. The Structure and Function I and II modules, as well as the summative exam format, remained consistent across the two academic years studied. No major changes were made to program structure, class content, or exam design.

### Clerkship Student Context

The M4 advanced anatomy elective was a 4-week course in which students assisted with prosection dissections, taught M1 laboratory sessions, and developed medical education resources, including questions in the style of National Board of Medical Examiners (NBME) exams. The elective ran five times (i.e., rotations) throughout the academic year and overlapped with the M1 courses noted above. Enrollment ranged from two to six students, depending on the rotation time frame selected and academic year. During the 2020–2021 academic year a total of 11 students enrolled in the elective, and 22 students enrolled during the 2021–2022 academic year. Students typically self-selected based on the anatomy content of interest (e.g., a student interested in cardiology might choose the elective running during the lower limb musculoskeletal and cardiopulmonary content). Baseline knowledge of M4 students was not formally assessed prior to participation; all students had previously completed the full anatomy curriculum during their first year and were in good academic standing. On the first day of the elective, students were introduced to NBME-style question writing and provided access to the NBME Gold Book, which outlines the question-writing process and provides examples [18]. At the end of each week, students participated in an NBME question workshop to review and refine questions. The elective was graded on a pass/fail basis based on anatomy faculty’s cumulative observations of student participation in all elective activities. Knowledge gained by peer-teachers was not directly measured as part of this study.

### M4 NBME Workshop Context

At the beginning of the elective, M4 students were introduced to NBME-style question writing and given access to the NBME Gold Book [18]. Each student was asked to create 3–5 questions individually each week based on the anatomy they were currently teaching. Time for question drafting was not formally scheduled; students were encouraged to complete questions outside of structured sessions. Students were instructed to draft questions independently without the use of automated tools. Because AI‑generated content was uncommon in academic settings at the time of data collection and no features suggestive of AI output were identified during review, AI use was considered unlikely, although it was not formally measured.

At the end of each week, M4s and faculty met for a 2-hour workshop to review the questions together. Minor edits, such as spelling, grammar, or abbreviation corrections, were addressed immediately during the session. If a question required substantial revision such as rewriting the vignette or modification of the distractors, the original author was asked to revise it for the following week. The number of questions requiring minor versus major revisions was not recorded.

All questions were reviewed and approved by both faculty and M4s before inclusion on an exam. A ratio of approximately one question written by an M4 to two questions written by faculty was targeted; however, final question selection for summative exams was determined by faculty based on lecture content and learning objectives. As a result, not all M4 questions were used, explaining the imperfect ratio (Table 1). This collaborative workshop format mirrors the process used by SOMG faculty, where all questions undergo peer review by colleagues familiar with the content. Finalized questions were uploaded to ExamSoft for use in summative exams. Questions authored by faculty and those authored by M4 students were presented in identical format on exams, with no codes or indicators to distinguish their source.

**Table 1:** Total number of questions per exam, number authored by faculty and M4 students, and percentage of student-generated questions included on each summative assessment. Values represent counts and percentages of M4-authored items used in each exam

Exam	Total # of exam questions	# of Anatomy clerkship faculty questions	# of Anatomy clerkship M4 student questions	% of Anatomy clerkship M4 student questions
Exam 1A	83	22	10	12%
Exam 2A	83	19	5	6%
Exam 1B	89	27	6	7%
Exam 2B	89	28	7	8%
Exam 1C	112	26	7	6%
Exam 2C	114	15	8	7%
Exam 1D	88	24	12	14%
Exam 2D	84	28	7	8%

### Outcome Measures

ExamSoft performance measures included item difficulty, discrimination index (DI), and point biserial. Item difficulty is calculated as the percentage of students who answered each test item correctly [19–21]. The degree to which a test item discriminates between high and low achieving students (DI=Higher_27_-Lower_27_; 27 = 27% high and low achieving students) is the discrimination index [20–22]. It is measured on a scale of -1 to 1 [22]. The higher the DI value associated with a test item, the better that item is at discriminating between students with high test scores from those with low test scores [22–25]. Point biserial assesses item quality correlation between the score on an item and the test score [22, 24, 26]. Like the discrimination index, point biserial ranges from − 1 to 1, with high values indicating “strong” items and low values indicating “weak” items [22, 26]. Generally, items with point biserials ≥ 0.2 are considered to have high correlation and positive association with overall performance on an assessment [22, 24, 26].

### Statistical Analysis

SFI and SFII exam data across the 2021-22 and 2022-23 academic years including average exam scores, item difficulty index, item point biserial, and item discrimination index were collected using ExamSoft and exported to Microsoft Excel 2021. Statistical analysis was performed using SPSS statistical package, version 27 and reported descriptively. Two-sample T tests with equal variances were employed to compare mean difficulty index, mean discrimination index, and point biserial between faculty-written and M4 student-written exam items across 8 exams.

## Results

Eight anatomy summative exams were given to M1 students during the 2 years of SFI and SFII modules studied (two during SFI and two during SFII each year). For each exam, the total number of questions, the number authored by faculty and by M4 students, and the percentage of student-generated questions were recorded and are presented in Table 1.

The item difficulty index, discrimination index, and point biserial were calculated for the questions written by faculty members and the questions written by M4 students. The means of these three measures were calculated for both groups. These values were then compared for each of the exams.

The mean difficulty index for exam questions written by faculty members did not differ significantly from that written by M4 students (Table 2).

**Table 2:** T-test for equality of means analysis of the mean difficulty index values for exams

Exam	Overall average anatomy difficulty index	Anatomy clerkship faculty mean difficulty index	Anatomy clerkship M4 student mean difficulty index	Two-sided *p*	Mean difference	Std. error Diff	CI Lower	CI Upper
Exam 1A	0.80105	0.7641	0.838	0.288	-0.07391	0.0684	-0.2136	0.06578
Exam 2A	0.8353	0.8735	0.7971	0.275	0.07632	0.06871	-0.06381	0.21645
Exam 1B	0.83595	0.7879	0.884	0.236	-0.09611	0.07881	-0.25954	0.06733
Exam 2B	0.86085	0.8467	0.875	0.676	-0.02833	0.06688	-0.16743	0.11076
Exam 1C	0.88025	0.8822	0.8783	0.942	0.00278	0.03777	-0.07426	0.07982
Exam 2C	0.86875	0.8525	0.885	0.478	-0.0325	0.04532	-0.12459	0.05959
Exam 1D	0.88285	0.8957	0.87	0.392	0.02571	0.02965	-0.03461	0.08604
Exam 2D	0.83285	0.86	0.8057	0.332	0.05429	0.05516	-0.05795	0.16652

The mean discrimination index for exam questions written by faculty members did not differ significantly from questions written by M4 students except for exam 2 C, where the questions written by M4 students had a mean discrimination index significantly higher than those written by faculty members (Table 3) (Exam 2 C: Faculty 0.8525, M4 0.8850, *P* = 0.031) (Table 4).

**Table 3:** T-test for equality of means analysis of the mean discrimination index values for exams

Exam	Overall average anatomy discrimination index	Anatomy clerkship faculty mean discrimination index	Anatomy clerkship M4 student mean discrimination index	Two-sided *p*	Mean difference	Std. error Diff	CI Lower	CI Upper
Exam 1A	0.2049	0.2227	0.187	0.542	0.03573	0.05793	-0.08259	0.15404
Exam 2A	0.2186	0.17	0.2671	0.183	-0.09714	0.07124	-0.24244	0.04816
Exam 1B	0.2167	0.2353	0.198	0.564	0.03726	0.06354	-0.09451	0.16903
Exam 2B	0.1738	0.2013	0.1463	0.389	0.05508	0.0626	-0.0751	0.18527
Exam 1C	0.1905	0.1644	0.2167	0.29	-0.05222	0.04856	-0.15125	0.04681
Exam 2C	0.1905	0.1467	0.2342	0.031	-0.0875	0.03889	-0.16654	-0.00846
Exam 1D	0.1764	0.1557	0.1971	0.439	-0.04143	0.05287	-0.149	0.06614
Exam 2D	0.2106	0.2554	0.1657	0.203	0.08964	0.08964	-0.05071	0.23

**Table 4:** T-test for equality of means analysis of the point biserial values for exams

Exam	Overall average anatomy point biseriasl	Anatomy clerkship faculty mean point biserial	Anatomy clerkship M4 student mean point biserial	Two-sided* p*	Mean difference	Diff error Std.	95% CI Lower	95% CI Upper
Exam 1A	0.2389	0.2418	0.236	0.908	0.00582	0.05	-0.09629	0.10793
Exam 2A	0.26605	0.255	0.2771	0.727	-0.02214	0.06285	-0.15033	0.10604
Exam 1B	0.252	0.26	0.244	0.768	0.016	0.05357	-0.0951	0.1271
Exam 2B	0.2287	0.236	0.2213	0.818	0.01475	0.06343	-0.11716	0.14666
Exam 1C	0.2519	0.2504	0.2533	0.962	-0.00296	0.06125	-0.12787	0.12195
Exam 2C	0.1905	0.1467	0.2342	0.001	-0.1725	0.04899	-0.27206	-0.07294
Exam 1D	0.2349	0.2268	0.2429	0.743	-0.01607	0.04863	-0.115	0.08286
Exam 2D	0.2565	0.33	0.1829	0.007	0.14714	0.0515	0.04236	0.25192

The mean point biserial for exam questions written by faculty members did not differ significantly from.

questions written by M4 students, except for exams 2 C and 2D. For exam 2 C, the questions written by M4 students had a higher point serial value than those written by faculty members. Conversely, the questions written by.

faculty members for exam 2D had a higher point serial value than those written by M4 students (Table 4). (Exam 2 C: Faculty 0.1467, M4 0.2342, *P* < 0.001; Exam 2D: Faculty 0.33, M4 0.1829, *P* = 0.007) (Table 4).

## Discussion

The present study sought to quantify the psychometric performance of exam questions written by M4 students in comparison to those written by faculty members in an ongoing discussion about the benefits of introducing medical education practice and assessment into the experiences of M4 students. Few significant differences were observed between the psychometric measures of anatomy exam questions authored by USCSOMG faculty and those authored by M4 students across two academic years. Mean item difficulty, mean discrimination index, and point biserial measures were largely comparable between the two groups for all 8 exams (Tables 1 and 2, and 3). The findings support both the null hypothesis presented in this paper and the findings of previous studies aiming to quantify the quality of instruction and assessment from M4 and faculty preceptors, indicating minimal differences between the two groups [7, 8].

The exceptions to this finding occurred in exams 2 C and 2D. The M4 cohort wrote items with a significantly higher mean discrimination index than faculty members for exam 2 C (Table 2) Conversely, anatomy faculty had significantly higher discrimination index and point biserial values for exam 2D (Tables 3 and 4).

Several factors may explain the discrepancies observed between exams 2 C and 2D. From a statistical standpoint, faculty consistently authored more questions than M4 students on all eight exams, although the ratio varied by exam. This imbalance was most pronounced in exam 2D which included only seven M4 authored items were included (Table 1). The smaller sample size may have exaggerated differences that would otherwise been negligible. In addition, the composition of the M4 students in each elective may also have influenced psychometric outcomes. While all M4 students had completed the full anatomy curriculum during their first year, individual familiarity with specific anatomical regions varied based on personal interest and intended specialty. For example, M4 students pursuing musculoskeletal specialties may be better prepared to write questions for exams focused on those regions than for an exam emphasizing cardiopulmonary anatomy. Because students were not assigned to elective rotations based on specialty interest, alignment between cohort expertise and exam content varied across electives. Given these factors, discrepancies between exams 2 C and 2D should be interpreted with caution.

A key limitation is that M4-authored and faculty-authored items were not used in their original form; all underwent faculty review and editing prior to implementation. Therefore, our findings reflect the performance of collaboratively refined items rather than purely student or faculty generated questions. The results may partly represent the effectiveness of faculty guidance in addition to student authorship.

Moreover, knowledge gained by peer-teachers through participation in NBME-style question writing was not directly measured. While the elective aimed to enhance both anatomical understanding and assessment literacy, future studies could incorporate pre- and post-intervention assessments or reflective surveys to evaluate these outcomes. In addition, time allocated for question drafting was not formally scheduled; students completed questions outside structured session, which may have contributed to variation in quality.

Another limitation is that the number of questions requiring minor versus major revisions was not recorded, restricting insight into the initial accuracy and completeness of student-authored items. Collecting these data in future iterations could help identify common challenges in question construction and inform targeted training. Finally, this study was conducted at a single institution with a voluntary elective cohort, which may limit generalizability. Findings may not apply to all fourth-year medical students or to students at other institutions.

Despite these limitations, the structured workshop format provided a collaborative environment that mirrored faculty practices and ensured rigorous peer review prior to exam inclusion. This study’s strength lies in its direct comparison of exam question quality metrics between faculty and M4 students. The different sample sizes for questions written by faculty versus M4 students, as well as varying levels of student familiarity with specific content, may have impacted the results. These findings suggest that M4 students can produce exam questions of comparable quality to those written by faculty. This underscores the potential benefits of integrating teaching and assessment responsibilities into the M4 curriculum. Having clinical students write NBME style clinical vignette-based multiple-choice questions for summative assessments allows these students to refresh their understanding of important concepts before entering residency and taking on the career-long responsibility of teaching medicine to students, junior faculty, and peer physicians. Further studies could explore the long-term benefits of peer-teaching on both M4 and pre-clerkship students and investigate how alignment of students’ specialty interests with the content they are tasked with teaching and assessing affects the quality of their questions.

## Conclusion

Medical education research has long championed the benefit of organizing learning environments and implementing programs for fourth-year medical students that emphasize teaching as a skill. Within this collaborative model, questions authored by M4 students and refined through faculty review performed similarly to faculty-authored items on key psychometric indices. These results should be interpreted with caution, as all student-generated questions underwent faculty editing prior to inclusion, meaning the findings reflect collaboratively refined items rather than purely student-authored content. Nevertheless, the comparable performance of these items suggests that integrating question-writing and teaching responsibilities into the M4 curriculum can be a valuable educational strategy. Future studies should explore outcomes using unedited student questions and examine the impact of faculty guidance on item quality.

## Data Availability

Data is available upon request and at the discretion of the authors.
